# Therapeutic Effects of Chinese Medicine Herb Pair, Huzhang and Guizhi, on Monosodium Urate Crystal-Induced Gouty Arthritis in Rats Revealed by Anti-Inflammatory Assessments and NMR-Based Metabonomics

**DOI:** 10.1155/2016/9398435

**Published:** 2016-02-16

**Authors:** Bin Han, Huizhu Huang, Zhong Li, Mengjuan Gong, Wan Shi, Chunxia Zhu, Zulian Gu, Zhongjie Zou

**Affiliations:** School of Traditional Chinese Medicine, Guangdong Pharmaceutical University, Guangzhou 510006, China

## Abstract

The present study was undertaken to evaluate the therapeutic effects of Huzhang-Guizhi herb pair (HG), firstly included in Hu-Zhang Power documented in Taiping Shenghui Fang, on monosodium urate (MSU) crystals-induced gouty arthritis in rats. We found that pretreatment with HG in rats with gouty arthritis could significantly attenuate the ankle joint swelling, and this beneficial antigout effect might be mediated, at least in part, by inhibiting tumor necrosis factor-alpha (TNF-*α*) and interleukin-1 beta (IL-1*β*) production in synovial fluid as well as nuclear transcription factor-*κ*B p65 (NF-*κ*B p65) protein expression in synovial tissue. Moreover, metabonomic analysis demonstrated that 5 and 6 potential biomarkers associated with gouty arthritis in plasma and urine, respectively, which were mainly involved in energy metabolism, amino acid metabolism, and gut microbe metabolism, were identified. HG could reverse the pathological process of MSU-induced gouty arthritis through regulating the disturbed metabolic pathways. These results provided important mechanistic insights into the protective effects of HG against MSU-induced gouty arthritis in rats.

## 1. Introduction

Gout, an ancient and common form of inflammatory arthritis, is a rheumatic disease resulting from deposition of uric acid crystals (monosodium urate, MSU) in tissues and fluids within the body. This process is caused by an overproduction or under excretion of uric acid. Certain common medications, alcohol, and dietary foods are known to be contributory factors. Acute gout will typically manifest itself as an acutely red, hot, and swollen joint with excruciating pain. These acute gouty flare-ups respond well to treatment with oral anti-inflammatory medicines and may be prevented with medication and dietary changes. Recurrent bouts of acute gout can lead to a degenerative form of chronic arthritis called gouty arthritis [1].

The goal of anti-inflammatory therapy for gouty arthritis is to eliminate the massive pain, swelling, and disability resulted from acute attacks [2]. Nonsteroidal anti-inflammatory drugs (NSAIDs) or colchicine is a first-line systemic treatment according to the current gouty arthritis treatment guidelines [3]. However, side effects of NSAIDs may include acute renal failure and gastrointestinal bleeding. Furthermore, treatment is contraindicated in patients with chronic kidney disease (CKD) [4]. As part of complementary and alternative medicine, traditional Chinese medication and acupuncture have also been used to treat gout, which have, in some circumstances, a superior side effect profile to NSAIDs [5–7].

Herb pairs (mixture of two herbs), as the basic composition units of Chinese herbal formulas, have special clinical significance in traditional Chinese medicine (TCM) and are much simpler than other complex formulas without altering their basic therapeutic features [8].* Polygonum cuspidatum* (known as “Hu-Zhang” in Chinese) has antiviral, antimicrobial, anti-inflammatory, neuroprotective, and cardioprotective functions [9].* Cassia twig* (known as “Gui Zhi” in Chinese) exhibits antiviral, antimicrobial, antineoplastic, anti-inflammatory, antiallergic, and analgesic properties [10]. The combination of these two herbs, known as Huzhang-Guizhi herb pair (HG) (with a ratio of 6 : 1), is firstly included in Hu-Zhang Power documented in Taiping Shenghui Fang, and our previous study demonstrated the antigout effects of HG in acute gouty arthritis rat model [11]. Meanwhile, HG is also used in other formulas, such as Lexing-tongfengke which has been widely used to treat gouty arthritis in clinics [12]. However, the biochemical mechanism by which HG acts in gouty arthritis still remains elusive. Our study, therefore, aims to elucidate the mechanisms by which HG acts therapeutically.

Metabonomics is a well-established field in systems biology and has been applied to observe meaningful and relevant biochemical changes due to disease, toxicity, nutrition, and other variables. Metabonomic studies can provide invaluable information towards understanding molecular mechanisms and novel insights into the status of dysfunction in biological systems [13]. Recently, using ultra-performance liquid chromatography coupled with mass spectrometry (UPLC/MS), the metabolic profiles of rats with MSU-induced gouty arthritis and the biological effects of acupuncture were investigated [5]. As we all know, both nuclear magnetic resonance (NMR) spectroscopy and mass spectrometry (MS) (usually with a chromatographic separation step) are suitable techniques for metabonomic analysis but have different analytical strengths and weaknesses and give complementary information [14]. To the best of our knowledge, metabonomic study of gouty arthritis in rat model based on NMR has not been reported.

In this study, we monitored intumesce index, histopathological changes, production of inflammatory cytokines, and immunohistochemistry of nuclear transcription factor-*κ*B p65 (NF-*κ*B p65) to investigate the efficacy and anti-inflammatory mechanism of HG against MSU crystal-induced gout in rats, an experimental model for gouty arthritis that is similar to those observed in clinical gout. Furthermore, a metabonomic method was applied to characterize the metabolic profiles associated with MSU-induced gouty arthritis and observe the protective effects of HG in rat plasma and urine.

## 2. Materials and Methods

### 2.1. Chemicals and Reagents

Huzhang and Guizhi were purchased from Heshuntang (Guangzhou, China) and authenticated by a specialist in pharmacognosy. Distilled water was purified using a Milli-Q ultrapure water system (Millipore, Bedford, MA, USA). MSU crystals and deuterium oxide (D_2_O, 99.9%) were obtained from Sigma-Aldrich (St. Louis, MO, USA). Rat tumor necrosis factor-alpha (TNF-*α*) and interleukin-1 beta (IL-1*β*) enzyme-linked immunosorbent assay (ELISA) kits were purchased from Affinity BioReagents (Golden, CO, USA). NF-*κ*B p65 antibody was purchased from Santa Cruz Biotechnology, Inc. (Santa Cruz, CA, USA).

### 2.2. Herbal Extraction

A mixture of Huzhang (60 g) and Guizhi (10 g) crushed into small pieces was extracted with water (1 : 10 w/v) under thermal reflux for 2 h. The filtrates were collected and the residues were then refluxed in water (1 : 10, w/v) for 1.5 h. Two batches of filtrates were combined and then were concentrated under a vacuum to obtain HG extract at a final concentration of 0.7 g/mL (w/v, expressed as the weight of raw materials).

### 2.3. Animal Handling and Sample Collection

All experiments were approved by the local ethics committee and all experimental procedures were in compliance with the National Institutes of Health Guide for Care and Use of Laboratory Animals.

A total of 18 adult male SD rats weighing 180–200 g were commercially obtained from Laboratory Animal Center of Traditional Chinese Medicine University of Guangzhou, China. Rats were maintained under controlled temperature (20 ± 2°C) and lighting (08:00–20:00) conditions with food and water available ad libitum. After 7 days of acclimatization, the animals were randomly divided into three groups (*n* = 6 in each group): (1) control group; (2) gouty arthritis model group, and (3) HG pretreatment group (HG group). Rats in the HG pretreatment group were administrated orally by gavage with HG at a dose of 9.3 g/kg of body weight once daily for consecutive 7 days. Dosing in this study was set according to dosages previously detailed in the literature [11]. The control and model groups received an equivalent volume of vehicle, in the form of sterile saline, as the pretreatment group for 7 days. Gouty arthritis was induced according to established protocol [15]: 1 h after the last dosing on day 7, MSU crystals (100 mg/mL, suspended in sterile saline) were injected in the left ankle joint of each rat in the model group and the HG group in a volume of 100 *μ*L. The control group received a 100 *μ*L intra-articular injection of single sterile saline. After the injection of MSU, urine was collected continuously (24 h) into ice-cooled vessels containing 0.5 mL of 2% sodium azide in metabolism cages. Following urine collection, all animals were anesthetized by diethyl ether inhalation and blood samples were acquired via the rats' orbital venous plexus. Heparin was used as an anticoagulant. Plasma was separated by centrifugation at 3500 rpm for 15 min. All urine and plasma samples were kept at −80°C until they were defrosted immediately prior to analysis. Synovial lavage fluid from each ankle was collected by injecting 5 mL phosphate buffered saline (PBS) into the joint cavity. Lavage fluids were then centrifuged at 400 g for 10 minutes and supernatants were stored at −80°C before biochemical determinations, and synovial tissue was gently separated for immunohistochemistry examination.

### 2.4. Measurement of Ankle Edema

The development of arthritis was assessed by measuring the ankle size before and after induction of gout. The circumference of the inflamed ankle joint of the animals was measured using a 3 mm wide tape measure without elasticity at 24 h after MSU crystal injection. In order to quantify ankle edema, the data were converted to a percentage of changes of ankle size as an intumesce index using the following formula: intumesce index = (measured ankle size − primary ankle size)/primary ankle size.

### 2.5. Measurement of Inflammatory Cytokines in the Ankle Joint Lavage Fluid

The levels of TNF-*α* and IL-1*β* in the ankle joint lavage fluid, which could be secreted during the process of acute gouty arthritis induced by MSU, were measured by using commercially available ELISA kits according to the manufacturer's instructions. The concentrations of IL-1*β* and TNF-*α* were calculated according to standard curves.

### 2.6. Histopathological Evaluation and Immunohistochemistry for NF-*κ*B p65

Synovial tissue was fixed in 10% formalin solution, dehydrated in ascending grades of alcohol, and embedded in paraffin. Paraffin sections were then cut to a thickness of 5 *μ*m and stained with hematoxylin and eosin (H&E) for histological evaluation according to standard procedures. The expression of NF-*κ*B p65 was investigated by immunohistochemistry analysis of 5 *μ*m paraffin sections. Briefly, sections of synovial tissues from different animal groups were deparaffinized with xylene and dehydrated in a graded ethanol series. Thereafter, tissue sections were microwaved for 8 min twice while immersed in 0.1 M citrate buffer (pH 6.0) to achieve antigen retrieval. After cooling, the sections were washed with PBS three times, and endogenous peroxidase activity was blocked with 3.0% H_2_O_2_ in methanol for 25 min at room temperature. After another three rinses with PBS, the tissues were blocked with 3% BSA (Sigma, St. Louis, MO, USA) for 30 min at room temperature and incubated with anti-NF-*κ*B p65 antibody (dilution 1 : 100) overnight at 4°C. After being washed three times in PBS, sections were incubated with goat anti-rat secondary antibody (Dako, CA, USA) at room temperature for 50 min. Following exposure to 3,3′-diaminobenzidine tetrahydrochloride (DAB), sections were counterstained with hematoxylin, dehydrated with increasing concentration of ethanol, and mounted with neutral gum. The slides were visualized under light microscope and the extent of cell immunopositivity was assessed.

### 2.7. Sample Preparation and ^1^H NMR Spectroscopy

400 *μ*L of rat urine was diluted with 200 *μ*L phosphate buffer (0.2 mol/L Na_2_HPO_4_-0.2 mol/L NaH_2_PO_4_, pH 7.4) thoroughly mixed in a vortex mixer and was subsequently centrifuged at 3500 rpm for 15 min at 4°C to remove sediment. 500 *μ*L of supernatant was transferred into a 5 mm NMR tube and 50 *μ*L of D_2_O (containing 0.05% W/V TSP-d_4_) was added. 400 *μ*L of plasma was transferred into a 5 mm NMR tube. Finally, 50 *μ*L of phosphate buffer (0.2 mol/L Na_2_HPO_4_-0.2 mol/L NaH_2_PO_4_, pH 7.4) and 50 *μ*L of D_2_O were mixed with the plasma. The D_2_O provided a field-frequency lock solvent for the NMR spectrometer [16].

^1^H-NMR spectra of the plasma and urine samples were recorded at 298 K on a Bruker AVANCE III 500 MHz spectrometer (BrukerBiospin, Rheinstetten, Germany) operating at 500.13 MHz ^1^H frequency. For plasma samples, the water-suppressed Carr-Purcell-Meibom-Gill (CPMG) spin-echo pulse sequence (RD-90°-(*τ*-180°-*τ*)*n*-ACQ) with a total spin-echo delay (2*nτ*) of 100 ms was used to attenuate broad signals from proteins and lipoproteins. 128 free induction decays (FIDs) were collected into 64 k data points over a spectral width of 10,000 Hz with a relaxation delay of 3 s and an acquisition time of 3.28 s. For urine samples, all ^1^H NMR spectra were collected using a standard 1D nuclear Overhauser enhancement spectroscopy- (NOESY-) presaturation pulse sequence. 128 free induction decays (FIDs) were collected into 64 k data points. Spectra were acquired with a spectral width of 10,000 Hz and an acquisition time of 3.28 s. Relaxation delay was set at 3 s.

### 2.8. Data Analysis

Data were expressed as mean ± standard deviation (SD). Statistical analysis was carried out using one-way analysis of variance (ANOVA) followed by post hoc Tukey's test. *P* values less than 0.05 were considered significant.

The obtained spectra of plasma and urine samples were processed with a 0.3 Hz line-broadening factor prior to Fourier transformation. All the spectra were manually corrected for phase and baseline distortions using MestReNova software (Mestrelab Research S.L, Santiago de Compostela, Spain). The spectra of plasma were referenced internally to methyl resonance of lactate (*δ*1.33). The plasma spectra were divided and the signal integral was computed in 0.01 intervals across the region *δ*0.50–9.0. The region of *δ*4.68–5.22 was removed to eliminate the effects of imperfect water saturation. The spectra of urine were referenced to the chemical shift of TSP at *δ*0. The spectra were divided and the signal integral computed in 0.01 intervals across the region *δ*0.5–9.0. The region of *δ*4.47–5.98 was removed to eliminate the influence of water and urea. Finally, all remaining regions of the spectra were then normalized to the total integrated area of the spectra to reduce any significant concentration differences.

The resultant integral data were imported into SIMCA-P 12.0 software (Umetrics, Umea, Sweden) for principal component analysis (PCA), partial least-squares discriminant analysis (PLS-DA), and orthogonal partial least-squares discriminant analysis (OPLS-DA) after mean-centering and pareto scaling, a technique that increased the importance of low abundance ions without significant amplification of noise. Model parameters, for example, goodness of fit, *R* ^2^, and goodness of predication, *Q* ^2^, were used to evaluate the quality of these models. Metabolites with VIP (variable importance in the projection) values ≥ 1.0 (obtained from OPLS-DA models) and values of *P* < 0.05 (calculated from independent sample *t*-test or Mann-Whitney *U*-test) are regarded as potential biomarkers [17].

## 3. Results

### 3.1. Effects of HG on Ankle Edema in MSU Crystal-Induced Gouty Arthritis Rats

**Figure 1:**
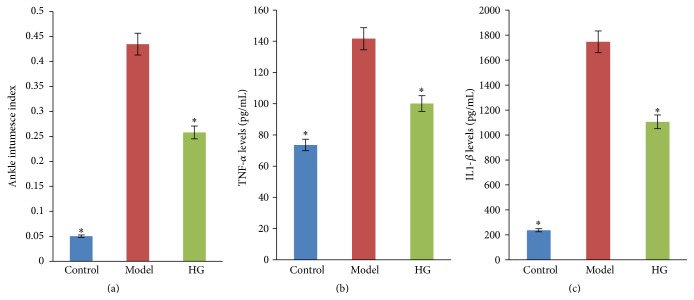
Effects of HG on ankle edema (a) and TNF-*α* (b) and IL-1*β* (c) production in the ankle joint lavage fluid in MSU crystal-induced gouty arthritis rats. ^*∗*^ *P* < 0.05 compared to the gouty arthritis model group.

As shown in Figure 1(a), there was a significant increase in the intumesce index in the gouty arthritis model group as compared to the control group (*P* < 0.05). HG could remarkably reduce the intumesce index in rats with MSU-induced gouty arthritis (*P* < 0.05).

### 3.2. Effects of HG on TNF-*α* and IL-1*β* Production in the Ankle Joint Lavage Fluid

The levels of TNF-*α* and IL-1*β* in the ankle joint lavage fluid were elevated markedly in the gouty arthritis model group when compared with the control group (*P* < 0.05, Figures 1(b) and 1(c)). HG-pretreated rats exhibited a significant reduction in all tested proinflammatory cytokines production in comparison with those in the gouty arthritis model group (*P* < 0.05).

### 3.3. Histopathological Effect of HG on Gouty Arthritis

**Figure 2:**
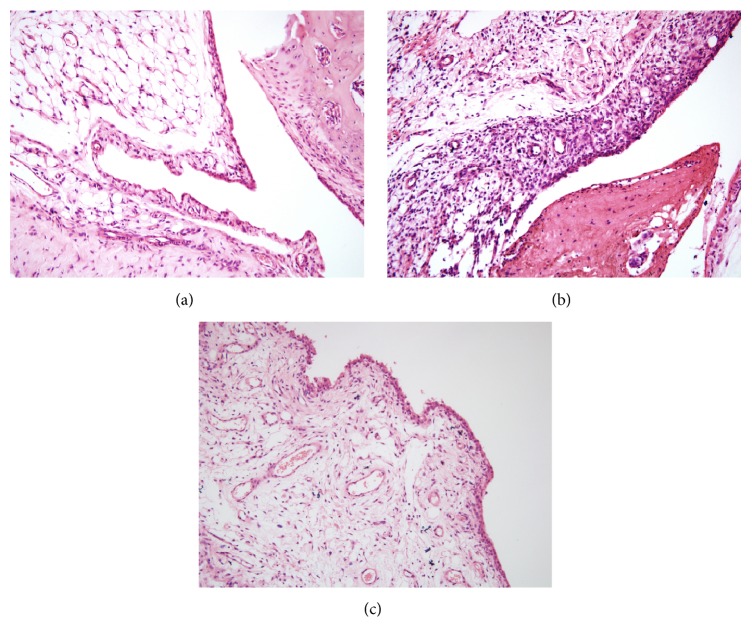
Effects of HG on synovial tissue histopathology of rats with gouty arthritis (magnification, ×200). (a) Control group, (b) model group, and (c) HG pretreatment group.

MSU crystals significantly increased leukocyte infiltration in the superficial and deeper layers of the synovium of ankle joint compared with those in control group and neutrophils were grossly abundant within the mixed cellular infiltrate in the synovium. The synovium became hyperplastic and thickened by inflammatory cells and formed a pannus that destroyed the underlying cartilage. In contrast, pretreatment with HG decreased leucocytes influx in the synovium of rats with gouty arthritis and slight hyperplasia was observed in synovial tissue (Figure 2).

### 3.4. Immunohistochemistry for NF-*κ*B p65

**Figure 3:**
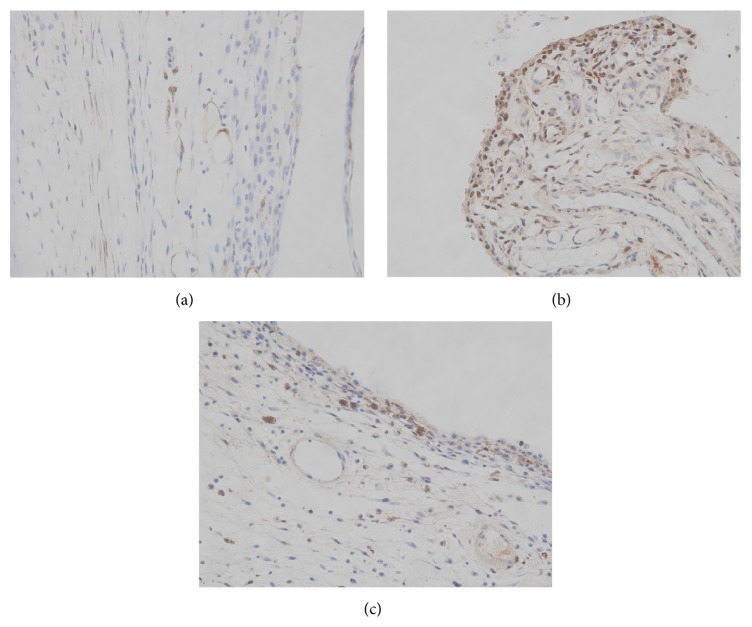
Effects of HG on the activation of NF-*κ*B p65 in synovial tissue of rats with gouty arthritis (magnification, ×400). (a) Control group, (b) model group, and (c) HG pretreatment group.

Compared to the control group, the positive expression of NF-*κ*B p65 proteins in the cytoplasm and the nucleus of synovium increased significantly in the gouty arthritis model group (*P* < 0.05). HG could inhibit NF-*κ*B p65 proteins overexpression induced by MSU crystals (Figure 3).

### 3.5. Analysis of ^1^H-NMR Profiles

**Figure 4:**
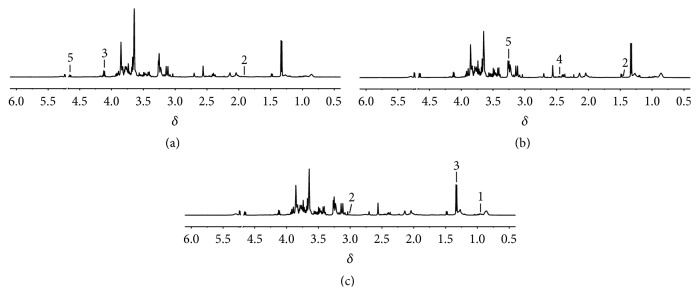
Representative ^1^H NMR spectra of rat plasma. Control (a), model (b), and HG pretreatment (c) groups. Keys: 1, leucine; 2, lysine; 3, lactate; 4, glutamine; 5, *β*-glucose.

**Figure 5:**
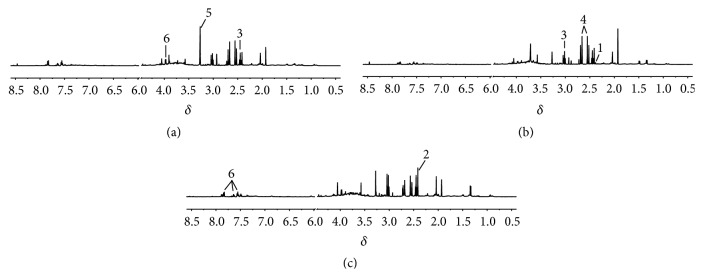
Representative ^1^H NMR spectra of rat urine. Control (a), model (b), and HG pretreatment (c) groups. Keys: 1, pyruvate; 2, succinate; 3, 2-oxoglutarate; 4, citrate; 5, trimethylamine N-oxide; 6, hippurate.

Typical ^1^H-NMR spectra obtained for plasma and urine were shown in Figures 4 and 5. The metabolite resonances were assigned according to previous studies [18–20] and the Chenomx NMR suite (Chenomx Inc., Edmonton, AB, Canada).

### 3.6. The Perturbation of Metabolic Profiles in Gout Model Rats

**Figure 6:**
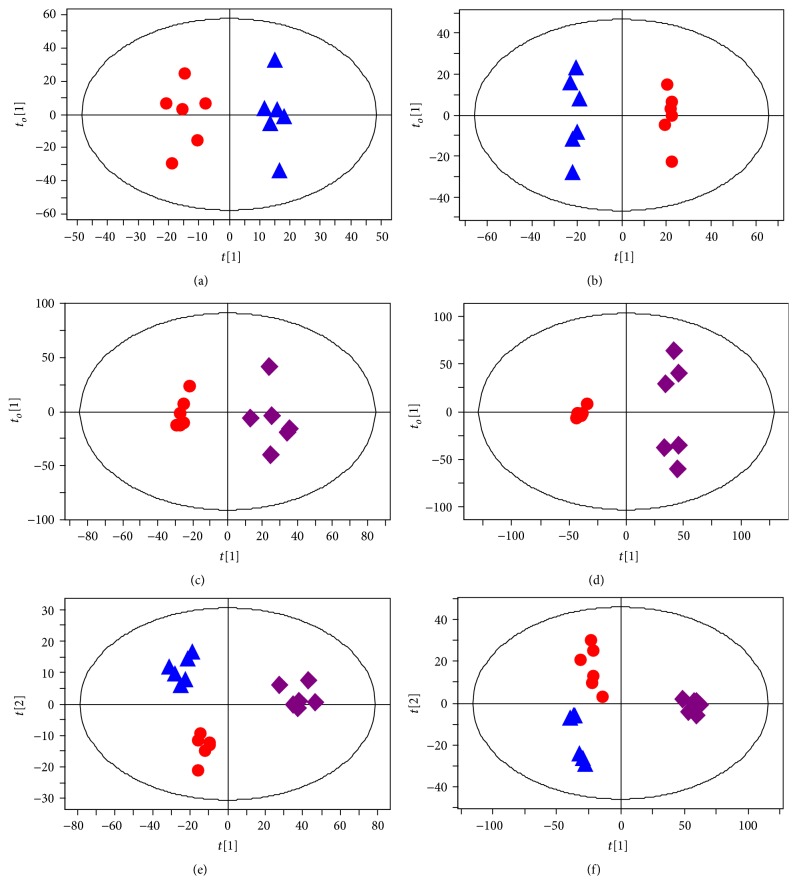
Metabolic profiles depicted by OPLS-DA score plots of ^1^H NMR spectral data of rat plasma ((a), (c), and (e)) and urine ((b), (d), and (f)) from control (▲, blue triangle), model (●, red dot), and HG pretreatment (*◆*, purple diamond) groups.

**Figure 7:**
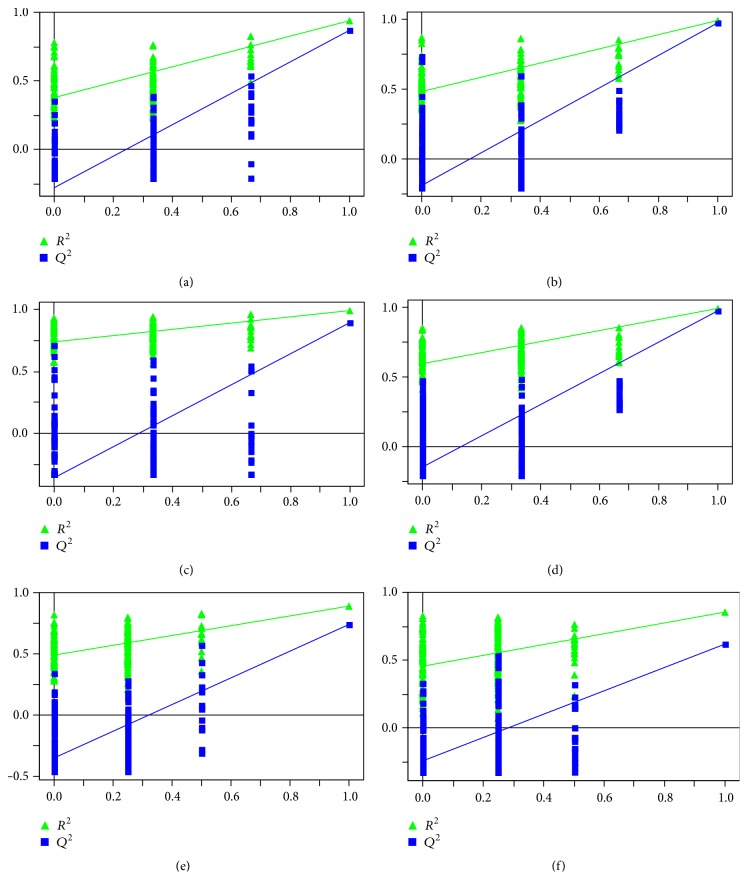
Statistical validation of the OPLS-DA models (Figures 6(a)–6(f)) by the corresponding PLS-DA models with the same number of components.

PCA and PLS-DA score plots were displayed in Supplementary Material available online at http://dx.doi.org/10.1155/2016/9398435 (Figures  1s and 2s). In order to maximize the separation between experimental groups and to focus on metabolic variations significantly contributing to classifications, the OPLS-DA models were subsequently performed. As shown in the OPLS-DA score plots (Figures 6(a) and 6(b)), a separation between the control and gouty arthritis model groups was clearly seen, indicating that they had completely different metabolic profiles. The model parameters were as follows: *R* ^2^ *X* = 0.836, *R* ^2^ *Y* = 0.949, and *Q* ^2^ = 0.832 for plasma; *R* ^2^ *X* = 0.871, *R* ^2^ *Y* = 0.996, and *Q* ^2^ = 0.859 for urine. In general, excellent models were obtained when values of *R* ^2^ *X*, *R* ^2^ *Y*, and *Q* ^2^ were above 0.8 [21]. Furthermore, to validate the robustness of these OPLS-DA models, 200-iteration permutation tests were performed in the corresponding PLS-DA models with the same number of components as the OPLS-DA models. The validation plots demonstrated that the original OPLS-DA models were not random and overfitted as both permutated *Q* ^2^ and *R* ^2^ values were significantly lower than the corresponding original values (Figures 7(a) and 7(b)) [16].

**Figure 8:**
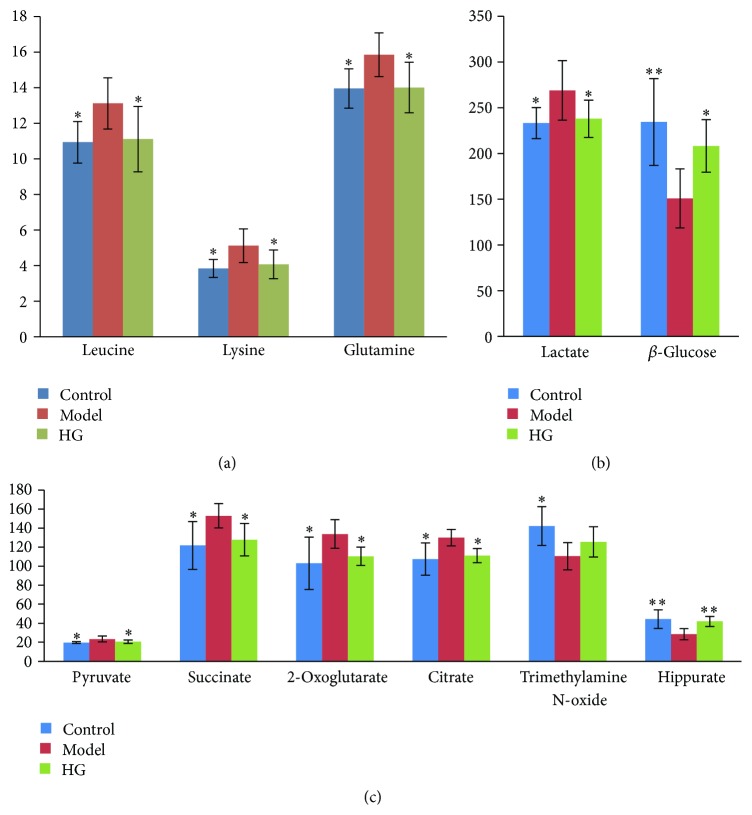
Bar charts of differentially expressed metabolite markers (mean ± SD, *n* = 6) in rat plasma ((a) and (b)) and urine (c). One-way ANOVA was used to determine the significance of difference. Compared to model group, *∗∗* and *∗* represented *P* < 0.01 and *P* < 0.05, respectively.

Selected according to the VIP values from the OPLS-DA models (VIP ≥ 1) and the *P* values from univariate statistical analysis (*P* < 0.05), 5 and 6 endogenous metabolites related to gouty arthritis were identified as potential biomarkers in plasma and urine, respectively (Figure 8 and Table 1). Compared to the control group, *β*-glucose was significantly downregulated, while leucine, lysine, lactate, and glutamine were significantly upregulated in the plasma of the gouty arthritis model group. The gouty arthritis model group also showed higher urinary levels of pyruvate, succinate, 2-oxoglutarate, and citrate, along with lower levels of trimethylamine N-oxide (TMAO) and hippurate when compared with the control group.

### 3.7. Intervention of HG on Metabolic Pattern of Rats with MSU-Induced Gouty Arthritis

The OPLS-DA scores plots constructed with NMR spectral data from rat plasma and urine samples in the control, model, and HG pretreatment groups showed a tendency recovering to healthy control group in HG pretreatment group as well as an obvious separation between gouty arthritis model group and HG pretreatment group (Figures 6(c)–6(f)). The corresponding validation plots (Figures 7(c)–7(f)) based on 200 times permutation tests demonstrated the robustness of these OPLS-DA models. In addition, all potential biomarkers associated with MSU-induced gouty arthritis in rats were significantly reversed by HG except trimethylamine N-oxide in urine (Figure 8 and Table 1). The above results, which were also in accordance with those in Figures 1–3, indicated that there was no doubt that pretreatment of HG in rats with gouty arthritis induced substantial and characteristic changes in their metabolic profiles.

## 4. Discussion

Gouty arthritis is an inflammatory response to MSU microcrystals that precipitate in joint tissues from supersaturated body fluids or are shed from preexisting articular deposits [22]. MSU crystals, first identified as the aetiological agent of gouty arthritis in the 18th century, can stimulate various types of cells, including monocytes macrophages, neutrophils, and synovial cells, resulting in a rapid increase in the production of proinflammatory cytokines and chemokines [7, 23, 24]. The clinical symptoms of inflammatory responses are characterized by severe pain, edema, and erythema in the joints. The potential for MSU crystal-induced rat joint inflammation as a valid experimental model of gouty arthritis was determined by testing classic antigout drugs [25, 26]. This experimental model caused a remarkable accumulation of neutrophils that was similar to the situation in humans. We used this well-established model to investigate the effects of HG on the arthritis induced by MSU crystals. The results demonstrated that HG significantly attenuated the swell in joint and reduced the intumesce index in rats with MSU-induced gouty arthritis. Furthermore, histopathological observations also showed that HG decreased leukocyte infiltration (primarily neutrophils) in the synovium of ankle joint in gouty arthritis rats. These results suggested that HG could protect animals from MSU crystal-induced gouty arthritis and joint injury.

TNF-*α* and IL-1*β* are thought to be particularly important in the pathogenesis of gouty arthritis [27, 28]. TNF-*α* and IL-1*β* mediate the rapid upregulation of the major endothelial ligand for neutrophils, E-selectin, in response to injection of MSU crystals into the joint space [27–30]. Both TNF-*α* and IL-1*β* are powerful stimuli for IL-6 and IL-8 productions, which can, respectively, account for the pronounced acute phase response [31, 32]. Here, we found that HG could significantly decrease the increased expression of TNF-*α* and IL-1*β* in rats with gouty arthritis.

NF-*κ*B is clearly one of the most important regulators of proinflammatory gene expression that can upregulate the expression of lots of cytokines such as TNF-*α*, IL-1*β*, and IL-8 [33]. The activated NF-*κ*B is reported to form a heterodimer, which usually consists of two proteins, p65 and p50 subunits. The p65 subunit has been demonstrated to exert critical activity on the transcription of many inflammatory genes, including adhesion molecules, cytokines, and chemokines [34]. Moreover, cytokines that are stimulated by NF-*κ*B, such as TNF-*α* and IL-1*β*, can also directly activate the NF-*κ*B pathway, thus establishing a positive autoregulatory loop that can amplify the inflammatory reaction [35]. Activation of NF-*κ*B may therefore be a key step in the pathogenesis of gouty arthritis, and suppression of NF-*κ*B is likely to be effective for the treatment of gouty arthritis. In the present study, immunohistochemistry analysis indicated that HG suppressed the expression of active NF-*κ*B p65 in the synovium.

Taken together, these findings suggested that the positive feedback loop between NF-*κ*B and extracellular signals including TNF-*α* and IL-1*β* might be interrupted by HG.

Changes in a number of metabolites involved in energy metabolism were observed in this work. The gouty arthritis model group showed a drop in glucose and elevated level of lactate in serum together with increased levels of succinate, 2-oxoglutarate, citrate, and pyruvate in urine as compared with the control group. Pyruvate, the product of glycolysis, represents an important junction point in carbohydrate catabolism under aerobic and anaerobic conditions. Citrate, 2-oxoglutarate, and succinate are key intermediate products of tricarboxylic acid (TCA) cycle which involves not only the glucose aerobic oxidation but also the major pathways for fat and amino acid metabolisms. Lactate is the end-product of glucose metabolism under anaerobic conditions. The elevated lactate was found to be a common and key biomarker of four types of human arthritis including gout [36]. Therefore, our results demonstrated the acceleration of glycolytic activity induced by gouty arthritis. The downregulation of succinate, 2-oxoglutarate, citrate, pyruvate, and lactate as well as upregulation of glucose was present in the HG pretreatment group compared with those in the model group, indicating that HG was able to efficaciously ameliorate the altered energy metabolism.

The metabolite profiling of plasma showed that leucine and lysine increased significantly in model rats, indicating gouty arthritis induced amino acid metabolism disturbance. Meanwhile, glutamine, the amide of glutamate, provides a nontoxic storage and transport form of ammonia. Thus, the altered level of glutamine in the model group might reflect disorders of glutamine formation and could also be used as an index of disturbance in amino acid metabolism caused by gouty arthritis. HG could restore the increased levels of leucine, lysine, and glutamine, manifesting its ability to rectify the disturbance of amino acid metabolism induced by gouty arthritis.

Urinary levels of TMAO and hippurate were significantly decreased in gouty arthritis model rats relative to the healthy controls. Reduced urinary excretion of hippurate was observed in patients with acute gout [37]. These metabolites are uniquely produced by bacterial metabolism in the intestinal tract, indicating that gouty arthritis may be associated with variations in intestinal microflora [38]. HG effectively attenuated the alteration of hippurate, reflecting its protective action on gut microbiota metabolism.

Although potential biomarkers associated with gouty arthritis were identified and intervention effects of HG were clearly observed, there were limitations in the current study. Firstly, owing to the lack of individual herb pretreatment groups, it was impossible to identify the role of each herb alone. It would be better to set five animal groups to compare the effects of each individual herb and the mixture in future study. Secondly, the underlying mechanisms of the observed changes in metabolic phenotype warrant further investigation with a goal to verify potential biomarkers in human afflicted with gouty arthritis and test new medications for their efficacy. Thirdly, additional mechanistic information and metabolite markers may be identified by using MS-based metabonomic techniques which provide complementary information to NMR.

## 5. Conclusions

In summary, antigout effects of HG were observed in rats with MSU-induced gouty arthritis, as evidenced by inhibition of joint swelling and suppression of inflammatory cell infiltration. This beneficial antigout effect might be mediated, at least in part, by inhibiting TNF-*α*, IL-1*β*, and NF-*κ*B p65 protein expression in synovial fluid and synovial tissue. In addition, ^1^H NMR-based metabonomic approach was firstly applied in this study to reveal that the pathophysiological process of MSU-induced gouty arthritis was closely related to disruption of several metabolic pathways including energy metabolism, amino acid metabolism, and gut microbe metabolism, and HG possessed protective effects against these metabolic dysfunctions. These results offered new insights into in-depth understanding of the pathogenesis of gouty arthritis, as well as discovery of targets for clinical diagnosis and treatment, and also provided further evidence for the potential use of HG as an antigout agent.

## Supplementary Material

In the Supplementary Material, metabolic profiles depicted by PCA and PLS-DA score plots of ^1^H NMR spectral data of rat plasma and urine from control, model and HG pretreatment groups were shown in Figures 1s and 2s, respectively.

## Figures and Tables

**Table 1:** Identification of significantly differential endogenous metabolites associated with gouty arthritis in rat plasma and urine.

Metabolite	Chemical shift (*δ*)^a^	VIP^b^	FC^c^	Control^d^	HG^d^
Plasma					
Leucine	0.96 (t)	1.6	1.20	↓^*∗*^	↓^*∗*^
Lysine	1.46 (m), 1.90 (m), 3.02 (m)	1.2	1.34	↓^*∗*^	↓^*∗*^
Lactate	1.33 (d), 4.12 (q)	3.1	1.15	↓^*∗*^	↓^*∗*^
Glutamine	2.45 (m), 3.77 (m)	1.5	1.14	↓^*∗*^	↓^*∗*^
*β*-Glucose	3.25 (dd), 4.65 (d)	4.5	0.64	↑^*∗∗*^	↑^*∗*^
Urine					
Pyruvate	2.38 (s)	1.3	1.19	↓^*∗*^	↓^*∗*^
Succinate	2.41 (s)	1.3	1.26	↓^*∗*^	↓^*∗*^
2-Oxoglutarate	2.45 (t), 3.02 (t)	1.7	1.30	↓^*∗*^	↓^*∗*^
Citrate	2.55 (d), 2.68 (d)	1.6	1.21	↓^*∗*^	↓^*∗*^
Trimethylamine N-oxide	3.28 (s)	2.1	0.78	↑^*∗*^	-
Hippurate	3.98 (d), 7.56 (t), 7.64 (t), 7.84 (d)	2.4	0.64	↑^*∗∗*^	↑^*∗∗*^

^a^Letters in parentheses indicate the peak multiplicities: s: singlet; d: doublet; t: triplet; q: quartet; m: multiplet.

^b^VIP was obtained from OPLS-DA model (Figure 6).

^c^Fold change (FC) was calculated as the ratio of the mean metabolite levels between model and control groups. FC with a value >1 indicates a relatively higher concentration while a value <1 means a relatively lower concentration present in model group as compared to the controls.

^d^Compared to model group, ↑ indicates relative increase in signal while ↓ indicates relative decrease in signal. *∗∗* and *∗* represent *P* < 0.01 and *P* < 0.05, respectively, whereas - denotes no statistically significant difference.
