# Supplementary material for: Therapeutic Effects of Chinese Medicine Herb Pair, Huzhang and Guizhi, on Monosodium Urate Crystal-Induced Gouty Arthritis in Rats Revealed by Anti-Inflammatory Assessments and NMR-Based Metabonomics
Source: Evid Based Complement Alternat Med. 2016 Feb 16;2016:9398435. doi: 10.1155/2016/9398435 (PMC4771918; doi:10.1155/2016/9398435)
Supplement: Supplementary file 1 — In the Supplementary Material, metabolic profiles depicted by PCA and PLS-DA score plots of 1H NMR spectral data of rat plasma and urine from control, model and HG pretreatment groups were shown in Figures 1s and 2s, respectively. [file 9398435.f1.doc]

**Therapeutic Effects of** **Chinese Medicine Herb Pair, Huzhang and Guizhi, on Monosodium Urate Crystal-induced Gouty Arthritis in Rats Revealed by Anti-inflammatory Assessments and NMR-Based Metabonomics**

Bin Han, Huizhu Huang, Zhong Li, Mengjuan Gong, Wan Shi, Chunxia Zhu, Zulian Gu, Zhongjie Zou

School of Traditional Chinese Medicine, Guangdong Pharmaceutical University, Guangzhou 510006, China

Correspondence should be addressed to Zhongjie Zou; zouzhongjie@139.com


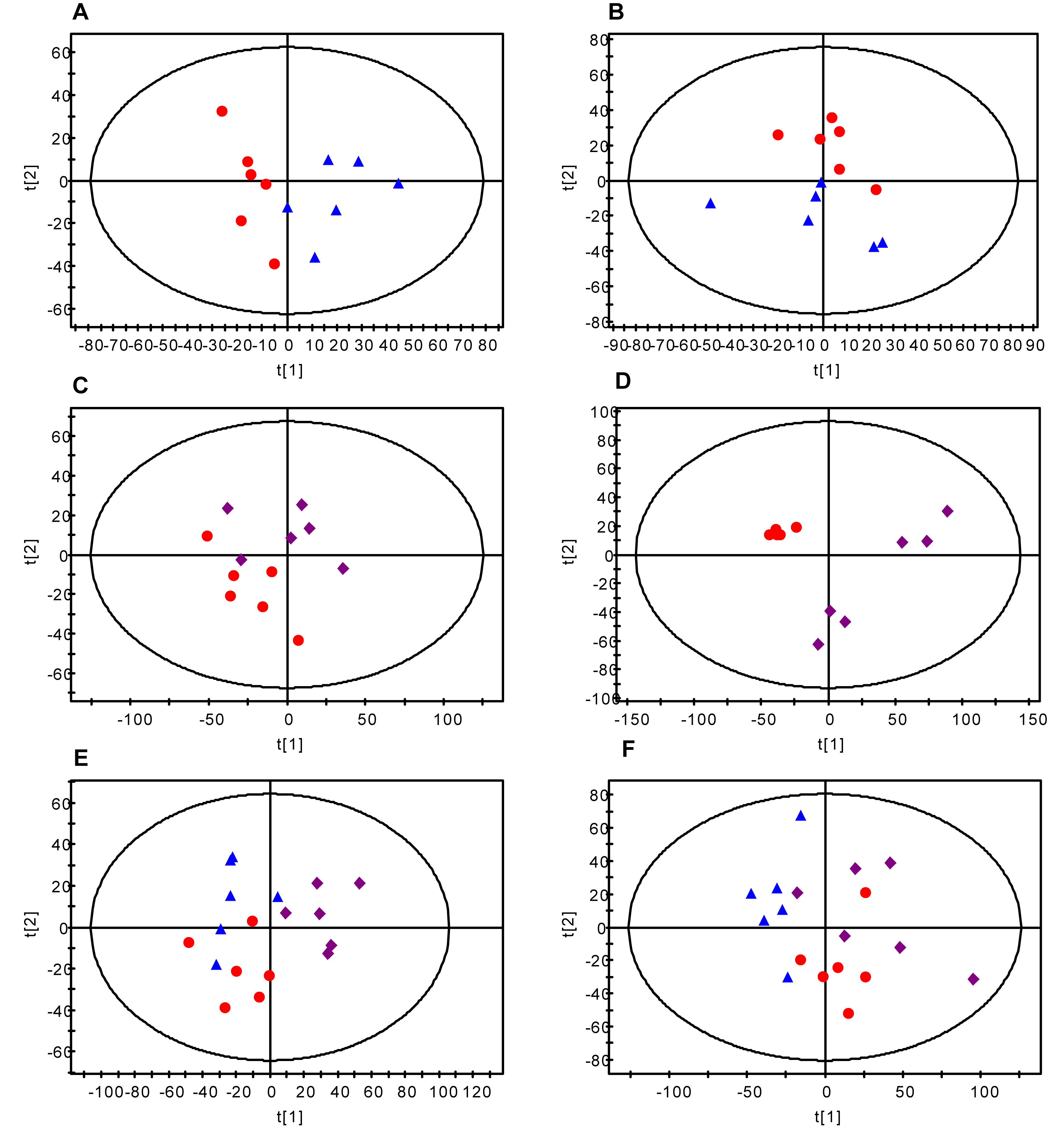


**Figure 1S** Metabolic profiles depicted by PCA score plots of ^1^H NMR spectral data of rat plasma (A, C and E) and urine (B, D and F) from control (▲, blue triangle), model (●, red dot) and HG pretreatment (♦, purple diamond) groups.


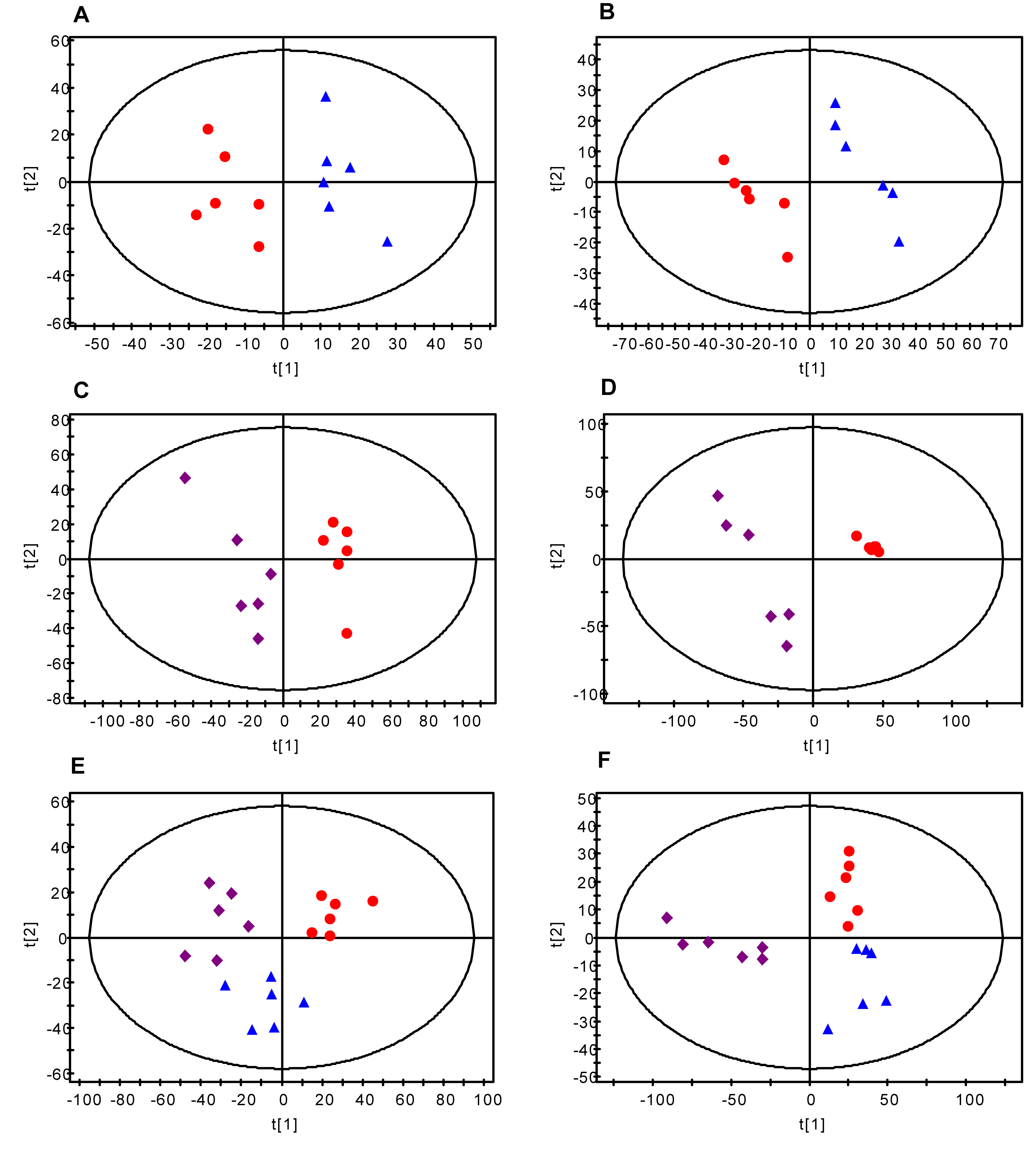


**Figure 2S** Metabolic profiles depicted by PLS-DA score plots of ^1^H NMR spectral data of rat plasma (A, C and E) and urine (B, D and F) from control (▲, blue triangle), model (●, red dot) and HG pretreatment (♦, purple diamond) groups.
